# P-1168. Activity of Isavuconazole and Comparator Agents against Pediatric Fungal Isolates Collected from 2017–2023 in a Global Surveillance Program

**DOI:** 10.1093/ofid/ofae631.1354

**Published:** 2025-01-29

**Authors:** Marisa Winkler, Paul Rhomberg, Kelley A Fedler, Abigail Klauer, Mariana Castanheira

**Affiliations:** Element Materials Technology/Jones Microbiology Institute, North Liberty, Iowa; Element Materials Technology/Jones Microbiology Institute, North Liberty, Iowa; Element, North Liberty, Iowa; Element Materials Technology/Jones Microbiology Institute, North Liberty, Iowa; JMI Laboratories, North Liberty, Iowa

## Abstract

**Background:**

Isavuconazole (ISA) is an azole antifungal agent with desirable properties, often making it the treatment of choice for invasive fungal infections. In December 2023, ISA became the sole FDA-approved azole antifungal for the treatment of invasive aspergillosis and mucormycosis in pediatric patients as young as 1 year old. Here, we compare the *in vitro* activity of ISA and comparator agents against pediatric fungal isolates collected from 2017–2023 in the SENTRY Antimicrobial Surveillance Program.

In vitro Activity of Isavuconazole and select comparator agents against pediatric isolates from the SENTRY database in 2017–2023

**Figure:**
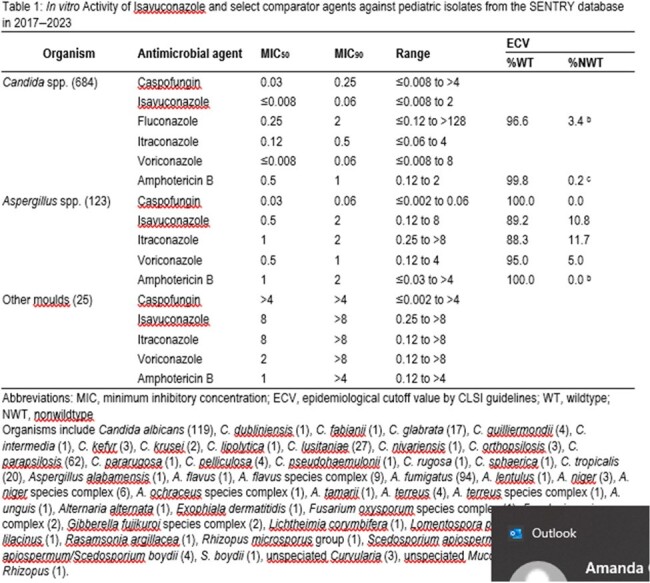

**Methods:**

A total of 851 isolates from invasive fungal infections were collected from patients ≤ 17 years old, including 284 patients ≤ 1 year old (yo), 186 2–5 yo, 204 6–12 yo, and 177 13–17 yo. The majority of isolates were from bloodstream infections (515/851, 60.5%). The second most common infection source was pneumonia in hospitalized patients (129/851, 15.2%). Antifungal susceptibility testing was performed by broth microdilution according to CLSI guidelines. Results were interpreted using epidemiological cutoff values (ECV) where applicable.

**Results:**

Isolates were from 28 different countries, with 25 hospitals in North America, 25 in Europe, 13 in Asia-Pacific, and 7 in Latin America. There were 684 *Candida* spp., 19 other yeasts, 123 *Aspergillus* spp., and 25 other moulds. For ISA tested against *Candida* spp., the MIC_50_ was ≤ 0.008 mg/L and the MIC_90_ was 0.06 mg/L (Table 1). This result was comparable to MIC_50/90_ for voriconazole (VRC) against *Candida* spp. The fluconazole (FLC) MIC_50/90_ was higher at 0.25/2 mg/L. For *Aspergillus* spp., the MIC_50/90_ for ISA was 0.5/2 mg/L and 89.2% of isolates were wildtype (WT) by ECV. The VRC MIC_50/90_ was 0.5/1 mg/L and 95% of isolates were WT. For Mucorales, MICs were 1–8 mg/L for ISA and 4– > 8 mg/L for VRC. For all other moulds, MICs ranged from 0.25– > 8 mg/L for ISA and 0.12– > 8 mg/L for VRC.

**Conclusion:**

ISA has good activity against pediatric fungal isolates from diverse geographical locations and infection sources. MICs were low for *Candida* spp. and *Aspergillus* spp., lower than FLC, and comparable to VRC. All agents had high MICs to non-*Aspergillus* moulds, highlighting the continued need for novel antifungal agents targeting these organisms.

**Disclosures:**

**Marisa Winkler, MD, PhD**, Element Iowa City (JMI Laboratories) was contracted to perform services in 2023 for > 30 biotech and pharmaceutical companies: Grant/Research Support **Kelley A. Fedler, BS**, Paratek Pharmaceuticals: Grant/Research Support

